# An Improved Feature Selection Based on Effective Range for Classification

**DOI:** 10.1155/2014/972125

**Published:** 2014-02-04

**Authors:** Jianzhong Wang, Shuang Zhou, Yugen Yi, Jun Kong

**Affiliations:** ^1^College of Computer Science and Information Technology, Northeast Normal University, Changchun 130000, China; ^2^National Engineering Laboratory for Druggable Gene and Protein Screening, Northeast Normal University, Changchun 130000, China; ^3^Key Laboratory of Intelligent Information Processing of Jilin Universities, Northeast Normal University, Changchun 130000, China; ^4^School of Mathematics and Statistics, Northeast Normal University, Changchun 130000, China

## Abstract

Feature selection is a key issue in the domain of machine learning and related fields. The results of feature selection can directly affect the classifier's classification accuracy and generalization performance. Recently, a statistical feature selection method named effective range based gene selection (ERGS) is proposed. However, ERGS only considers the overlapping area (OA) among effective ranges of each class for every feature; it fails to handle the problem of the inclusion relation of effective ranges. In order to overcome this limitation, a novel efficient statistical feature selection approach called improved feature selection based on effective range (IFSER) is proposed in this paper. In IFSER, an including area (IA) is introduced to characterize the inclusion relation of effective ranges. Moreover, the samples' proportion for each feature of every class in both OA and IA is also taken into consideration. Therefore, IFSER outperforms the original ERGS and some other state-of-the-art algorithms. Experiments on several well-known databases are performed to demonstrate the effectiveness of the proposed method.

## 1. Introduction

Feature selection is widely used in the domain of pattern recognition, image processing, data mining, and machine learning before the tasks of clustering, classification, recognition, and mining [1]. In real-world applications, the huge dataset usually has a large number of features which contains much irrelevant or redundant information [1]. Redundant and irrelevant features cannot improve the learning accuracy and even deteriorate the performance of the learning models. Therefore, selecting an appropriate and small feature subset from the original features not only helps to overcome the “curse of dimensionality” but also contributes to accomplish the learning tasks effectively [2]. The aim of feature selection is to find a feature subset that has the most discriminative information from the original feature set. In general, feature selection methods are usually divided into three categories: embedded, wrapper, and filter methods [3, 4]. They are categorized based on whether or not they are combined with a specific learning algorithm.

In the embedded methods, the feature selection algorithm is always regarded as a component in the learning model. The most typical embedded based feature selection algorithms are decision tree approaches, such as ID3 [5], C4.5 [6], and CART algorithm [7]. In these algorithms, the features with the strongest ability of classification are selected in the nodes of the tree, and then the selected features are utilized to conduct a subspace to perform the learning tasks. Obviously the process of decision tree generation is also feature selection process.

Wrapper methods directly use the selected features to train a specific classifier and evaluate the selected subset according to the performance of the classifier. Therefore, the performances of wrapper methods strongly depend on the given classifier. Sequential forward selection (SFS) and sequential backward selection (SBS) [8] are two well-studied wrapper methods. SFS was initialized to an empty set. Then, the best feature from the complete feature set was chosen according to the evaluation criteria in each step and added into the candidate feature subset until it meets the stop condition. On the contrary, SBS started from the complete feature set. Then, it eliminated a feature which has the minimal impact on the classifier in each step until it satisfied the stop condition. Recently, Kabir et al. proposed a new wrapper based feature selection approach using neural network [9]. The algorithm was called constructive approach for feature selection (CAFS). The algorithm used a constructive approach involving correlation information to select the features and determine the architectures of neural network. Another wrapper based feature selection method was also proposed by Ye and Gong. In their approach, they considered the feature subset as the evaluation unit and the subset's convergence ability was utilized as the evaluation standard [10] for feature selection.

Different from the embedded and wrapper based algorithms, filter based feature selection methods directly select the best feature subset based on the intrinsic properties of the data. Therefore, the process of feature selection and learning model is independent in them. At present, the algorithms of filter based feature selection can be divided into two classes [11]: ranking and space searching. For the former, the feature selection process can be regarded as a ranking problem. More specifically, the weight (or score) of each feature is firstly computed. Then, the top *k* features are selected according to the ascending order of weight (or score). Pearson Correlation Coefficient (PCC) [12], Mutual Information (MI) [13], and Information Gain (IG) [14] are three commonly used ranking criterion to measure the dependency between each feature and the target variable. Another ranking criterion method named Relief [15], which analyzed the importance of each feature by computing the relationship between an instance and its nearest neighbors from the same and different classes, was proposed by Kira and Rendell. Then, an extension of Relief termed Relief-F was developed in [16]. Besides, there also exist many other methods proposed for ranking based filter feature selection. For more details about these algorithms, the readers can refer to [3, 4]. Although the ranking based filter methods have been applied to some real-world tasks successfully, a common shortcoming of these methods is that the feature subset selected by them may contain redundancy. In order to solve this problem, some space searching based filter methods have been proposed to remove the redundancy during feature selection. Correlation-based feature selection (CFS) [17] is a typical space searching algorithm; it did not only consider the correlation among features but also take the correlation between features and classes into account. Thus, CFS inclined to select the subset contains features that are highly correlated with the class and uncorrelated with each other. Minimum redundancy maximum relevance (MRMR) [18] is another method presented to reduce the redundancy of the selected feature subset.

Since both embedded and wrapper based feature selection methods interact with the classifier, they can only select the optimal subset for a particular classifier. So the features selected by them may be worse for other classifiers. Moreover, another disadvantage of the two methods is that they are more time consuming than filter method. Therefore, filter method is more fit for dealing with data that has large amounts of features since it has a good generalization ability [19]. As a result, we mainly focus on the research for filter based feature selection in this work.

In this paper, an integrated algorithm named Improved feature selection based on effective range (IFSER) is proposed for filter based feature selection. Our IFSER can be considered as an extension of the study in [20]. In [20], Chandra and Gupta presented a new statistical feature selection method named effective range based gene selection (ERGS). ERGS utilized the effective range of statistical inference theory [21] to calculate the weight of each feature, and a higher weight was assigned to the most important feature to distinguish different classes. However, since ERGS only considered the overlapping area (OA) among effective range of each class for every feature, it fails to handle the other relationships among the features of different classes. In order to overcome this limitation, the concept of including area (IA) is introduced into the proposed IFSER to characterize the inclusion relationship of effective ranges. Moreover, the samples' proportion for each feature of every class in both OA and IA is also taken into consideration in our IFSER. Therefore, IFSER outperforms the original ERGS and some other state-of-the-art algorithms. Experiments on several well-known databases are performed to demonstrate the effectiveness of the proposed method.

The rest of this paper is organized as follows.  Section 2 briefly reviews ERGS and effective range. The proposed IFSER is introduced in Section 3.  Section 4 reports experimental results on four datasets. Finally, we provide some conclusions in Section 5.

## 2. A Briefly Review on ERGS

In this section, we will review the effective range and ERGS algorithm briefly [20].

Let *F* = {*F*_*i*_} be the feature set of the dataset *X* ∈ *R* ^*N*×*d*^,  *i* = 1,2,…, *d*. *Y* = {*Y*_*j*_}  (*j* = 1, 2,…, *l*) is the class labels of *X*. The class probability of *j*th class *Y*_*j*_ is *p*_*j*_. For each class *Y*_*j*_ of the *i*th feature *F*_*i*_, *μ*_*ij*_ and *σ*_*ij*_ denote the mean and standard deviation of the *i*th feature *F*_*i*_ for class *Y*_*j*_, respectively. Effective range (*R*_*ij*_) of *j*th class *Y*_*j*_ for *i*th feature *F*_*i*_ is defined by

(1) $$\begin{array}{c} R_{ij}=\left[r_{ij}^{-},r_{ij}^{+}\right]=\left[\mu_{ij}-\left(1-p_{j}\right)\gamma \sigma_{ij},\mu_{ij}+\left(1-p_{j}\right)\gamma \sigma_{ij}\right], \end{array}$$

where *r*_*ij*_^−^ and *r*_*ij*_^+^ are the lower and upper bounds of the effective range, respectively. The prior probability of *j*th class is *p*_*j*_. Here, the factor (1 − *p*_*j*_) is taken to scale down effect of class with high probabilities and consequently large variance. The value of *γ* is determined statistically by Chebyshev inequality defined as

(2) $$\begin{array}{c} P\left(\left|X-\mu_{ij}\right|\geq \gamma \sigma_{ij}\right)\leq \frac{1}{\gamma^{2}} \end{array}$$

which is true for all distributions. The value of *γ* is set as 1.732 for the effective range which contains at least 2/3rd of the data objects [20].

Overlapping area (OA_*i*_) among classes of feature *F*_*i*_ is computed by

(3) $$\begin{array}{c} \text{O}{\text{A}}_{i}=\sum_{j=1}^{l-1}\sum_{k=j+1}^{l}\varphi_{i}\left(j,k\right), \end{array}$$

where *φ*_*i*_(*j*, *k*) can be defined as

(4) $$\begin{array}{c} \varphi_{i}\left(j,k\right)=\{\begin{array}{cc} r_{ij}^{+}-r_{ik}^{-} & \text{if}\;\;r_{ij}^{+}>r_{ik}^{-}\\ 0 & \text{otherwise}. \end{array} \end{array}$$

In ERGS, for a given feature, the effective range of every class is first calculated. Then, the overlapping area of the effective ranges is calculated according to (3), and the area coefficient is computed for each feature. Next, the normalized area coefficient is regarded as the weight for every feature and an appropriate number of features are selected on the basis of feature weight. For more detailed information about the ERGS algorithm, the readers can refer to [20].

## 3. Improved Feature Selection Based on Effective Range

In this section, we present our improved feature selection based on effective range (IFSER) algorithm, which integrates overlapping area, including area and the samples' proportion for each feature of every class, into a unified feature selection framework.

### 3.1. Motivation

**Figure 1:**
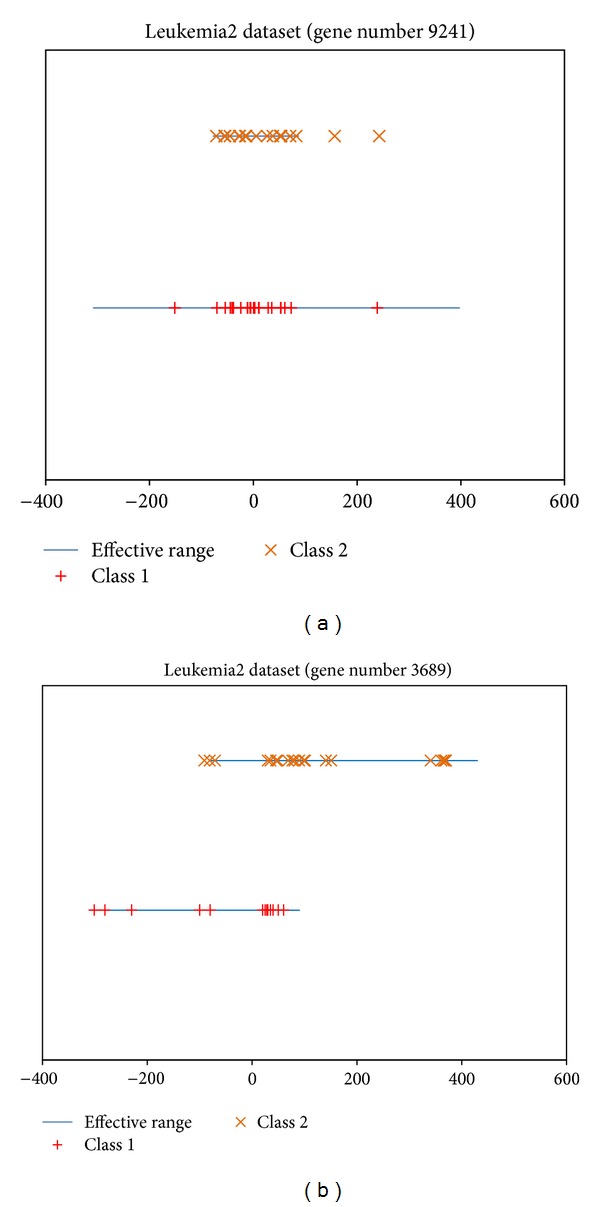
The ER of the gene accessions numbers 9241 and 3689 from the Leukemia2 gene database.

Although ERGS considers the overlapping area of every class for each feature, it fails to handle the problem of the inclusion relation of effective ranges. The problem is very realistic in real-world applications. Taking the gene data set as an example, Figure 1 shows the effective ranges of two gene samples from the Leukemia2 [22] gene database. From this figure, we can see that the overlapping area of gene number 9241 in Figure 1(a) is 165.7, and the overlapping area of gene number 3689 in Figure 1(b) is 170.8. Since the two overlapping areas of these two genes are similar, their weights obtained by ERGS are also similar. However, the relationships between the effective ranges in these two genes are very different. In Figure 1(a), the effective range of class 1 is completely included in the effective range of class 2, while the effective range of class 1 is partly overlapping with the effective range of class 2 in Figure 1(b). Therefore, the weight of the gene number 9241 in Figure 1(a) should be less than that in Figure 1(b) since all the samples in class 1 cannot be corrected and classified in this case. For this reason, the inclusion relation between the effective ranges (including area) must be taken into consideration.

**Figure 2:**
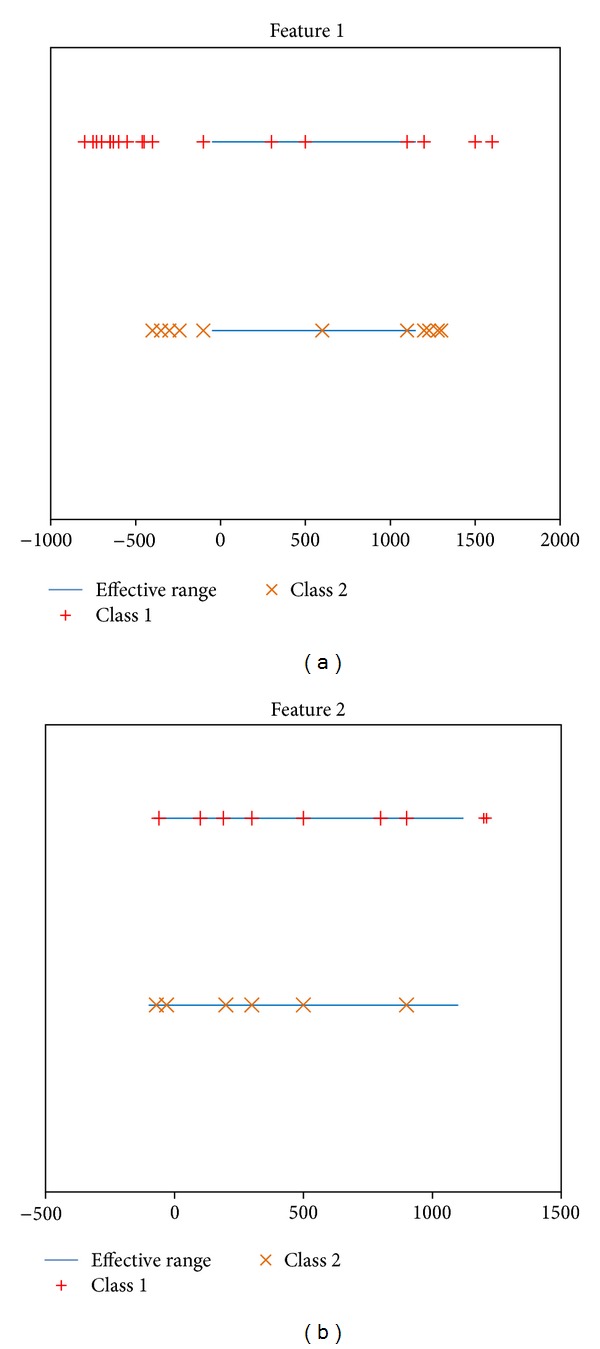
Different feature with the same size of overlapping area but different sample proportions in the two areas.

Another example is shown in Figure 2. As can be seen from this figure, it is clearly found that the two features in Figures 2(a) and 2(b) have the same size of the overlapping area. However, the number of samples in these two areas is very different. In Figure 2(a), the number of samples belonging to the overlapping area is small but the number of samples belonging to the overlapping area in Figure 2(b) is relatively large. Thus, it is obvious that feature 1 is more important than feature 2 since more samples can be correctly classified. In other words, the weight assigned to feature1 should be greater than that assigned to feature 2. From this example, we can see that the samples' proportion for each feature of every class in both overlapping and including areas is also a vital factor to influence the features' weights and should be considered in the feature selection process.

### 3.2. Improved Feature Selection Based on Effective Range

Similar to ERGS, we suppose *F* = {*F*_*i*_} is the feature set of the dataset *X* ∈ *R* ^*N*×*d*^, *i* = 1,2,…, *d*. *Y* = {*Y*_*j*_}  (*j* = 1,2,…, *l*) is the class label set of the data samples in *X*. The class probability of *j*th class *Y*_*j*_ is *p*_*j*_. For each class *Y*_*j*_ of *i*th feature *F*_*i*_, *μ*_*ij*_ and *σ*_*ij*_ denote the mean and standard deviation of the *i*th feature *F*_*i*_ in class *Y*_*j*_, respectively.

The first step of our proposed IFSER is to calculate the effective range of every class by

(5) $$\begin{array}{c} R_{ij}=\left[r_{ij}^{-},r_{ij}^{+}\right]=\left[\mu_{ij}-\left(1-p_{j}\right)\gamma \sigma_{ij},\mu_{ij}+\left(1-p_{j}\right)\gamma \sigma_{ij}\right], \end{array}$$

where the definitions of *r*_*ij*_^−^, *r*_*ij*_^+^, *p*_*j*_, and 1 − *p*_*j*_ are the same as those in ERGS.

The second step of our IFSER is to calculate overlapping areas OA_*i*_ among classes of feature *F*_*i*_  (*i* = 1,2,…, *d*) by

(6) $$\begin{array}{c} {\text{OA}}_{i}=\sum_{j=1}^{l-1}\sum_{k=j+1}^{l}\varphi_{i}\left(j,k\right), \end{array}$$

where the definition of *φ*_*i*_(*j*, *k*) is as same as in ERGS.

The third step of our proposed IFSER is to compute including area IA_*i*_ among classes of feature *F*_*i*_  (*i* = 1,2,…, *d*) by

(7) $$\begin{array}{c} {\text{IA}}_{i}=\sum_{j=1}^{l-1}\sum_{k=j+1}^{l}\psi_{i}\left(j,k\right), \end{array}$$

where *ψ*_*i*_(*j*, *k*) can be defined as

(8) $$\begin{array}{c} \psi_{i}\left(j,k\right)=\{\begin{array}{cc} r_{ik}^{+}-r_{ik}^{-} & \text{if}\;\;r_{ij}^{+}\geq r_{ik}^{+}\\ 0 & \text{otherwise}. \end{array} \end{array}$$

The fourth step of our proposed IFSER is to compute area coefficient (AC_*i*_) of feature *F*_*i*_  (*i* = 1,2,…, *d*) as

(9) $$\begin{array}{c} \text{A}{\text{C}}_{i}=\frac{\text{S}{\text{A}}_{i}}{\text{Ma}{\text{x}}_{j}\left(r_{ij}^{+}\right)-\text{Mi}{\text{n}}_{j}\left(r_{ij}^{-}\right)}, \end{array}$$

where SA_*i*_ = OA_*i*_ + IA_*i*_. Then, the normalized area coefficient (NAC_*i*_) can be obtained by

(10) $$\begin{array}{c} \text{NA}{\text{C}}_{i}=1-\frac{\text{A}{\text{C}}_{i}}{\max\left(\text{A}{\text{C}}_{s}\right)},\;\text{for}\;s=1,2,\ldots ,d. \end{array}$$

From (10), we can clearly see that the features with larger NAC values are more important for distinguishing different classes.

The fifth step of our proposed IFSER is to calculate the samples' number of each class in OA_*i*_ and IA_*i*_ for each feature *F*_*i*_. Let *H*_*ij*_ and *G*_*ij*_ denote samples' numbers of the *j*th class in OA_*i*_ and IA_*i*_ for each feature *F*_*i*_. Assume that  *K*_*j*_ represents the number of samples in the*j*th class. Then we use *K*_*j*_ divided by *H*_*ij*_ and *G*_*ij*_ to represent the proportions of samples in OA_*i*_ and IA_*i*_, and for all classes of each feature the sums of the *H*_*ij*_/*K*_*j*_ and *G*_*ij*_/*K*_*j*_ are written as *H*_*i*_ and *G*_*i*_.

For all classes of each feature *F*_*i*_, the normalized *H*_*i*_ and *G*_*i*_ can be obtained by

(11) $$\begin{array}{c} \begin{array}{c} NH_{i}=1-\frac{H_{i}}{\max\left(H_{s}\right)}\\ GH_{i}=1-\frac{G_{i}}{\max\left(G_{s}\right)}, \end{array}\;\text{for}\;s=1,2,\ldots ,d. \end{array}$$

From (11), the larger the value of *NH*_*i*_ and *GH*_*i*_, the more significant the feature is.

The last step of our proposed IFSER is to compute the weight of each feature as

(12) $$\begin{array}{c} W_{i}=V_{i}\times Z_{i}, \end{array}$$

where *V*_*i*_ = NAC_*i*_ and *Z*_*i*_ = *NH*_*i*_ + *GH*_*i*_. From (12), we can see that a larger value of *W*_*i*_ indicates that the *i*th feature is more important. Therefore, we can select the features according to their weights and choose features with larger weights to form the selected feature subset.

Finally, the proposed IFSER algorithm can be summarized as in Algorithm 1.

## 4. Experiment and Results

In this section, in order to verify the performance of our proposed method, we conducted experiments on four datasets (Lymphoma [23], Leukemia1 [24], Leukemia2 [22], and 9_Tumors [25]) and compare our algorithm with five popular feature selection algorithms including ERGS [20], PCC [12], Relief-F [16], MRMR [18], and Information Gain [14]. Three classifiers are used to verify the effectiveness of our proposed method. The classification accuracies are obtained through leave-one-out cross-validation (LOOCV) in this work.

### 4.1. The Description of Datasets

#### 4.1.1. Lymphoma Database

The Lymphoma database [23] consists of 96 samples and 4026 genes. There are two classes of samples in the dataset. The dataset comes from a study on diffuse large B-cell lymphoma.

#### 4.1.2. Leukemia1 Database

Leukemia1 database [24] contains three types of Leukemia samples. The database has been constructed from 72 people who have acute myelogenous leukemia (AML), acute lymphoblastic leukemia (ALL) B cell, or ALL T-cell, and each sample is composed of 5327 gene expression profiles.

#### 4.1.3. Leukemia2 Database

The Leukemia2 dataset [22] contains a total of 72 samples in three classes: AML, ALL, and mixed-lineage leukemia (MLL). The number of genes is 11225.

#### 4.1.4. 9_Tumors Database

9_Tumors database [25] consists of 60 samples of 5726 genes and categorized into 9 various human tumor types.

### 4.2. Experimental Results Using C4.5 Classifier

In this subsection, we estimate the performance of our proposed IFSER using C4.5 classifier on the four gene databases. Tables 1, 2, 3, and 4 summarize the results of the classification accuracies achieved by our methods and other methods. As we can see from Tables 1–4, the proposed IFSER method performs better than the other five algorithms in most cases. In particular, our proposed IFSER is much better than ERGS. The reason is that our proposed IFSER not only considers the overlapping area (OA) but also takes the including area and samples' proportion into account. These results demonstrate the fact that IFSER is able to select the best informative genes compared to other well-known techniques.

For Lymphoma database, the classification accuracy of our proposed IFSER is substantial improvement compared with other algorithms. What is more, it is worth mentioning that our method only uses 10 features to achieve 93.75% classification accuracy. With the increase in feature dimension, the classification results of most methods (such as our proposed IFSER, PCC, IG, and ERGS) are reduced. For Relief-F and MRMR, the classification results are very low when the feature dimension is equal to 10 at the beginning. Then, with the increase in feature dimension, the classification results are improved. When they achieve the best results, the classification results begin to decrease with the increase in the dimension again.

For Leukemia1 and Leukemia2 databases, the performance of our proposed IFSER is also better than ERGS and other methods. Our proposed IFSER can achieve the best results when the feature dimension is between 50 and 70. For Leukemia1 database, the performances of MRMR and ERGS keep stable on most dimensions. The trend of the classification results of PCC on Leukemia2 is inconsistent with those on Lymphoma database since it is almost monotonously decreased with the increase of feature dimension. And the other results are consistent with the experiments on Lymphoma database.

For 9_Tumors database, as we can see from Table 4, the performances of all the methods are very low due to the fact that database only contains 60 samples but 5726 genes. However, the performance of our proposed IFSER is much better than other algorithms. This result demonstrates the fact that our proposed IFSER is able to deal with the small sample size and high dimensions gene data.

### 4.3. Experimental Results Using NN Classifier

In this subsection, we evaluate the performance of our proposed IFSER using nearest neighbor (NN) classifier on the four gene databases. The results of the classification accuracies achieved by our proposed and other methods are listed in Tables 5, 6, 7, and 8. Comparing Tables 5–8 with Tables 1–4, we can see that the classification results of all the methods are improved. For Lymphoma database, IFSER, PCC, and ERGS are better than Relief-F, IG, and MRMR. For Leukemia1 database, our proposed IFSER and PCC outperform Relief-F, IG, MRMR, and ERGS. And the best result of IFSER is the same as PCC. However, for Leukemia2, IFSER, IG, and Relief-F achieve the best results than PCC, MRMR, and ERGS. For 9_Tumors database, the performance of IFSER is worse than PCC, IG, and MRMR, but better than Relief-F and ERGS. These results demonstrate the fact that result of feature selection depends on the classifier, and it is crucial to choose an appropriate classifier for different feature selection methods.

### 4.4. Experimental Results Using SVM Classifier

**Figure 3:**
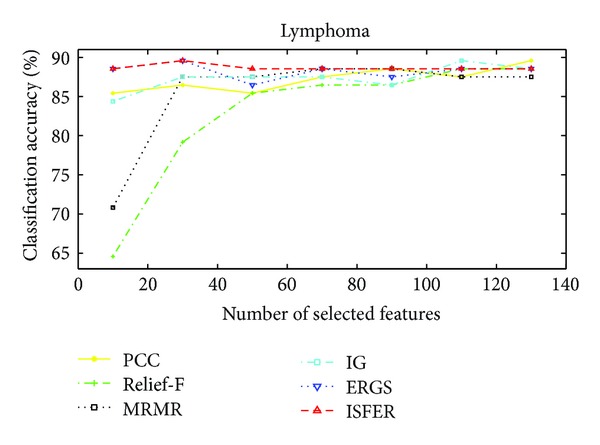
The classification accuracies of different algorithms on the Lymphoma database.

**Figure 4:**
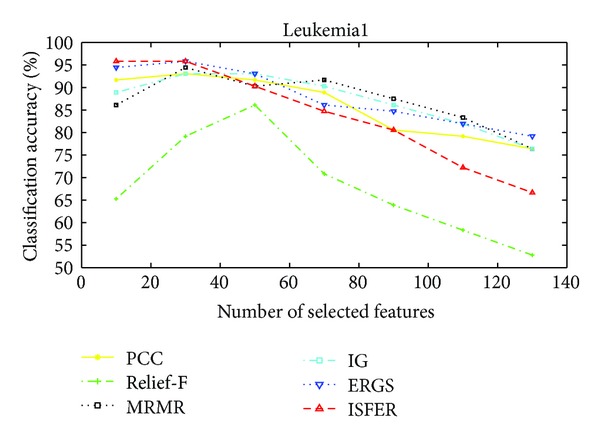
The classification accuracies of different algorithms on the Leukemia1 database.

**Figure 5:**
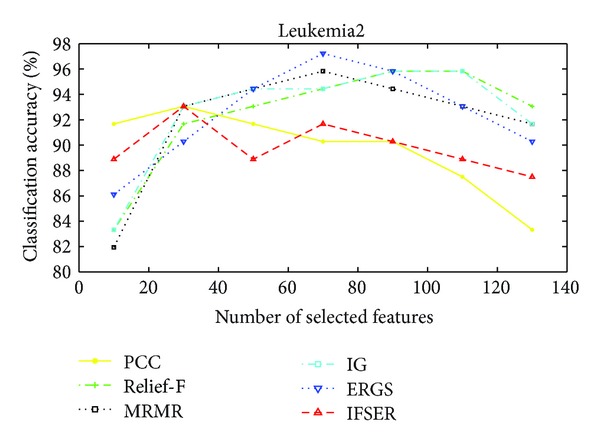
The classification accuracies of different algorithms on the Leukemia2 database.

**Figure 6:**
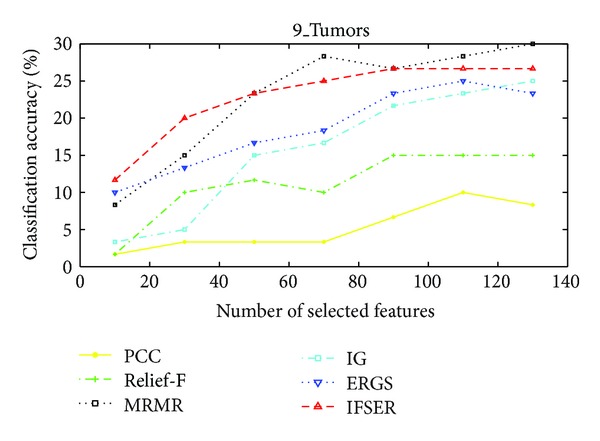
The classification accuracies of different algorithms on the 9_Tumors database.

The performance of our proposed IFSER using support vector machine (SVM) classifier on the four gene database is tested in this subsection. Figures 3–6 show the classification accuracies of different algorithms on four gene databases. From Figures 3 and 4, we can see that our proposed IFSER outperforms other algorithms in most cases. And the IFSER achieves its best result at a lower dimension than other algorithms. This result further demonstrates the fact that IFSER is able to select the best informative genes as compared to other feature selection techniques. As we can see from Figure 5, our proposed IFSER is worse than Relief-F, IG, MRMR and ERGS. From Figure 6, it is found that our proposed IFSRE outperforms PPC, Relief-F, IG, and ERGS but is not as good as MRMR. This indicates that the SVM classifier is not suitable for the feature selected results of our proposed algorithm on small sample size databases.

## 5. Conclusions

In this paper, we propose a novel statistical feature selection algorithm named effective range based gene selection (IFSER). Compared with existing algorithms, IFSER not only considers the overlapping areas of the features in different classes but also takes the including areas and the samples' proportion in overlapping and including areas into account. Therefore, IFSER outperforms the original ERGS and some other state-of-the-art algorithms. Experiments on several well-known databases are performed to demonstrate the effectiveness of the proposed method.

## Figures and Tables

**Algorithm 1:**
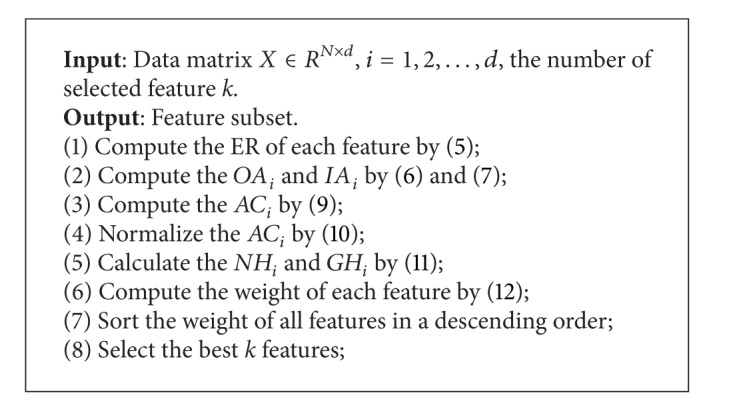


**Table 1:** Classification accuracies (%) of different feature selection methods with C4.5 on Lymphoma database.

	10	30	50	70	90	110	130
PCC	84.38	82.29	80.21	78.13	79.17	79.17	80.21
Relief-F	69.79	72.92	72.92	75.00	68.75	66.67	82.29
IG	78.13	76.04	76.04	72.92	77.08	77.08	77.08
MRMR	71.88	79.17	79.17	80.21	81.25	80.21	79.17
ERGS	86.46	85.42	82.29	81.25	83.33	83.33	84.38
IFSER	93.75	86.46	83.33	83.33	83.33	80.21	79.17

**Table 2:** Classification accuracies (%) of different feature selection methods with C4.5 on Leukemia1 database.

	10	30	50	70	90	110	130
PCC	88.89	88.89	88.89	87.50	87.50	87.50	87.50
Relief-F	75.00	79.17	75.00	80.56	81.94	79.17	80.56
IG	80.56	84.72	84.72	84.72	84.72	84.72	84.72
MRMR	84.72	84.72	84.72	84.72	84.72	84.72	84.72
ERGS	88.89	88.89	88.89	88.89	88.89	88.89	88.89
IFSER	84.72	84.72	86.11	90.28	90.28	88.89	87.50

**Table 3:** Classification accuracies (%) of different feature selection methods with C4.5 on Leukemia2 database.

	10	30	50	70	90	110	130
PCC	80.56	83.33	87.50	87.50	87.50	86.11	86.11
Relief-F	77.78	75.00	84.72	86.11	80.56	77.78	76.39
IG	84.72	87.50	87.50	87.50	87.50	87.50	87.50
MRMR	84.72	88.89	88.89	88.89	88.89	88.89	88.89
ERGS	86.11	84.72	88.89	88.89	88.89	87.50	87.50
IFSER	79.17	88.89	90.28	88.89	88.89	87.50	88.89

**Table 4:** Classification accuracies (%) of different feature selection methods with C4.5 on 9_Tumors database.

	10	30	50	70	90	110	130
PCC	28.33	28.33	26.67	25.00	28.33	26.67	28.33
Relief-F	20.00	16.67	30.00	28.33	31.67	36.67	36.67
IG	38.33	38.33	41.67	40.00	40.00	40.00	38.33
MRMR	38.33	38.33	40.00	36.67	38.33	40.00	40.00
ERGS	28.33	28.33	23.33	25.00	23.33	21.67	26.67
IFSER	25.00	36.67	43.33	48.33	46.67	43.33	43.33

**Table 5:** Classification accuracies (%) of different feature selection methods with NN on Lymphoma database.

	10	30	50	70	90	110	130
PCC	89.58	96.88	94.79	95.83	97.92	97.92	96.88
Relief-F	68.75	84.38	86.46	88.54	87.50	85.42	88.54
IG	88.54	95.83	94.79	94.79	95.83	96.88	96.88
MRMR	88.54	91.67	93.75	93.75	93.75	93.75	93.75
ERGS	89.58	94.79	95.83	97.92	95.83	97.92	97.92
IFSER	94.79	94.79	96.88	96.88	97.92	97.92	97.92

**Table 6:** Classification accuracies (%) of different feature selection methods with NN on Leukemia1 database.

	10	30	50	70	90	110	130
PCC	93.06	94.44	95.83	97.22	95.83	97.22	95.83
Relief-F	69.44	76.31	75.00	75.00	73.61	76.39	80.56
IG	93.06	94.44	91.67	93.06	93.06	94.44	93.06
MRMR	88.89	93.06	90.28	93.06	93.06	94.44	93.06
ERGS	94.44	95.83	94.44	95.83	95.83	95.83	95.83
IFSER	81.94	91.67	93.06	91.67	97.22	94.44	95.83

**Table 7:** Classification accuracies (%) of different feature selection methods with NN on Leukemia2 database.

	10	30	50	70	90	110	130
PCC	88.89	88.89	90.28	93.06	91.67	91.67	91.67
Relief-F	69.44	83.33	83.33	83.33	87.50	93.06	94.44
IG	83.33	83.33	94.44	94.44	94.44	94.44	94.44
MRMR	88.89	90.28	93.06	93.06	93.06	93.06	93.06
ERGS	86.11	86.11	93.06	93.06	91.67	93.06	93.06
IFSER	84.27	91.67	93.06	91.67	88.89	90.28	94.44

**Table 8:** Classification accuracies (%) of different feature selection methods with NN on 9_Tumors database.

	10	30	50	70	90	110	130
PCC	28.33	41.67	51.67	51.67	51.67	50.00	51.67
Relief-F	25.00	28.33	21.67	26.67	30.00	35.00	33.33
IG	48.33	51.67	60.00	58.33	60.00	61.67	58.33
MRMR	38.33	46.67	56.67	55.00	60.00	65.00	61.67
ERGS	25.00	30.00	40.00	38.33	41.67	41.67	45.00
IFSER	35.00	36.67	38.33	46.67	46.67	45.00	46.67

## References

[1] Xing E, Jordan M, Karp R (2005). Feature selection algorithms for classification and clustering. *IEEE Transactions on Knowledge and Data Engineering*.

[2] Guyon I, Elisseeff A (2003). An introduction to variable and feature selection. *Journal of Machine Learning Research*.

[3] Saeys Y, Inza I, Larrañaga P (2007). A review of feature selection techniques in bioinformatics. *Bioinformatics*.

[4] Lazar C, Taminau J, Meganck S (2012). A survey on filter techniques for feature selection in gene expression microarray analysis. *IEEE/ACM Transactions on Computational Biology and Bioinformatics*.

[5] Quinlan JR (1983). Learning efficient classification procedures and their application to chess end games. *Machine Learning: An Artificial Intelligence Approach*.

[6] Quinlan JR (1993). *C4.5: Programs For Machine Learning*.

[7] Breiman L, Friedman JH (1984). *Classification and Regression Trees*.

[8] Kittler J, Chen CH (1978). Feature set search algorithms. *Pattern Recognition and Signal Processing*.

[9] Kabir MM, Islam MM, Murase K (2010). A new wrapper feature selection approach using neural network. *Neurocomputing*.

[10] Ye JX, Gong XL (2010). A novel fast Wrapper for feature subset selection. *Journal of Changsha University of Science and Technology*.

[11] Wang J, Wu L, Kong J, Li Y, Zhang B (2013). Maximum weight and minimum redundancy: a novel framework for feature subset selection. *Pattern Recognition*.

[12] Van’t Veer LJ, Dai H, Van de Vijver MJ (2002). Gene expression profiling predicts clinical outcome of breast cancer. *Nature*.

[13] Peng H, Long F, Ding C (2005). Feature selection based on mutual information: criteria of max-dependency, Max-relevance, and Min-redundancy. *IEEE Transactions on Pattern Analysis and Machine Intelligence*.

[14] Liu H, Li J, Wong L (2002). A comparative study on feature selection and classification methods using gene expression profiles and proteomic patterns. *Genome Informatics Series*.

[15] Kira K, Rendell LA A practical approach to feature selection.

[16] Kononenko I Estimating features: analysis and extension of RELIEF.

[17] Hall MA (1999). *Correlation-based feature selection for machine learning [Ph.D. thesis]*.

[18] Ding C, Peng H (2005). Minimum redundancy feature selection from microarray gene expression data. *Journal of Bioinformatics and Computational Biology*.

[19] Almuallim H, Dietterich T Learning with many irrelevant features.

[20] Chandra B, Gupta M (2011). An efficient statistical feature selection approach for classification of gene expression data. *Journal of Biomedical Informatics*.

[21] Härdle W, Simar L (2007). *Applied Multivariate Statistical Analysis*.

[22] Armstrong SA, Staunton JE, Silverman LB (2002). *MLL* translocations specify a distinct gene expression profile that distinguishes a unique leukemia. *Nature Genetics*.

[23] Alizadeh A (2000). Distinct types of diffuse large B-cell lymphoma identified by gene expression profiling. *Nature*.

[24] Chemosensitivity prediction by transcriptional profiling *Whitehead Massachusetts Institute of Technology Center For Genome Research*.

[25] Golub TR, Slonim DK, Tamayo P (1999). Molecular classification of cancer: class discovery and class prediction by gene expression monitoring. *Science*.

